# Clinical and radiological outcomes of longCOVID: Is the post-COVID fibrosis common?

**DOI:** 10.5578/tt.20239907

**Published:** 2023-03-10

**Authors:** N SARIOĞLU, GD AKSU, H ÇOBAN, E BÜLBÜL, G DEMİRPOLAT, AT ARSLAN

**Affiliations:** 1 Department of Pulmonology, Balıkesir University Faculty of Medicine, Balıkesir, Türkiye; 2 Department of Radiology, Balıkesir University Faculty of Medicine, Balıkesir, Türkiye

**Keywords:** Post-COVID syndrome, dyspnea, fibrotic like changes

## Abstract

**ABSTRACT:**

Clinical and radiological outcomes of long-COVID: Is the post-COVID fibrosis common?

**Introduction:**

COVID-19 survivors may take longer to regain full well-being. This study aimed to investigate clinical and functional evaluation and radiologic changes in the third month after COVID-19.

**Materials and Methods:**

A total of 126 patients were assessed in the third month for symptoms, pulmonary function, exercise capacity, radiologic imaging, and quality of life after being discharged following COVID-19 treatment. Two radiologists evaluated the initial and follow-up images.

**Results:**

At the third month follow-up visit, the most common persisting symptoms were shortness of breath (32.5%), cough (12.7%), and muscle pain (12.7%). At the follow-up visit, oxygen saturations at rest and after a sixmin walking test were lower in patients with prior intensive care hospitalization compared to those without (p< 0.001, p= 0.004). Computed tomography (CT) scans revealed persisting pulmonary pathologies in 64.6% of patients at the third month follow-up. The most common pathologies on follow-up thoracic CT were fibrotic-like changes in 44.2% and ground-glass opacities (GGO) in 33.3%. Regression analysis unveiled that age [95% confidence interval (CI), 1.01 to 1.15; p= 0.020], male sex (95% CI, 4.06 to 95.3, p< 0.001), first CT severity score (95% CI, 1.02 to 1.41, p= 0.028), duration of hospitalization (95% CI, 1.02 to 1.18, p= 0.012), oxygen saturation (95% CI, 0.86 to 0.96, p< 0.001) were independent predictors of fibrotic-like changes.

**Conclusion:**

In the third month following COVID-19, the most common symptom was dyspnea, and the most common radiological findings were fibrotic-like changes and GGO. Longer follow-up studies of COVID-19 survivors are needed to observe lasting changes.

## Abstract (tr)

**ÖZ:**

Long-COVID klinik ve radyolojik sonuçlar: Post-COVID fibrozis yaygın mıdır?

**Giriş:**

COVID-19 geçirenlerde tam iyilik haline kavuşma uzun sürebilmektedir. Bu çalışmada COVID-19 sonrası üçüncü ayda klinik ve fonksiyonel değerlendirme ve radyolojik değişikliklerin incelenmesi amaçlanmıştır.

**Materyal ve Metod:**

COVID-19 tanısıyla taburcu edilen ve üçüncü ay kontrol vizitine gelen 126 hasta semptomlar, solunum fonksiyonları, egzersiz kapasitesi, radyolojik görüntüleme ve yaşam kalitesi ile değerlendirilmiştir. İki radyolog tarafından ilk ve kontrol görüntülemeleri karşılaştırarak incelenmiştir.

**Bulgular:**

Üçüncü ay kontrol vizitinde en sık devam eden semptomlar, nefes darlığı (32,5%), öksürük (12,7%) ve kas ağrısı (12,7%) idi. Öncesinde yoğun bakım yatışı olan hastaların kontrol vizitinde, istirahatte ve altı dk yürüme sonrası bakılan oksijen satürasyonları yoğun bakım yatışı bulunmayanlara göre daha düşüktü (p< 0,001, p= 0,004). Bilgisayarlı tomografi incelemeleri üçüncü ayda hastaların %64,6’sında radyolojik patolojilerin devam ettiğini ortaya koydu. Kontrol toraks bilgisayarlı tomografide en sık gözlenen bulgular %44,2’sinde fibrotik benzeri değişiklikler ve %33,3’ünde buzlu cam opasiteleri (GGO) idi. Yaş, [%95 güven aralığı (CI), 1,01 ile 1,15; p= 0,020], erkek cinsiyet (%95 GA, 4,06 ile 95,3; p< 0,001), ilk BT şiddet skoru (95% GA, 1,02 ile 1,41; p= 0,028), hastanede yatış süresi (%95 GA, 1,02 ile 1,18; p= 0,012), oksijen satürasyonu (%95 GA, 0,86 ile 0,96; p< 0,001) fibrotik benzeri değişiklikler için bağımsız belirleyiciler olarak saptandı.

**Sonuç:**

COVID-19 sonrası üçüncü ayda en sık görülen semptom nefes darlığı, en sık görülen radyolojik bulgu ise fibrotik benzeri değişiklikler ve buzlu cam opasiteleridir. Kalıcı değişiklikleri gözlemlemek için COVID-19 geçirenlerde daha uzun süreli takip çalışmalarına ihtiyaç vardır.

## Introduction

Coronavirus disease-2019 (COVID-19) has been a global pandemic since its identification, and its impacts remain ongoing. According to a report published by the World Health Organization on August 5, 2022, approximately 6.4 million deaths have been recorded worldwide ( 1 ).

Many people who have had COVID-19 had varying experiences with the disease, and the terms postCOVID and long-COVID are now more widely used to describe its long-term effects. In reality, however, the long-term effects of COVID-19 remain unknown.

Dyspnea, cough, hypoxemia, and ground-glass opacities were detected radiologically in individuals requiring hospitalization for acute disease ( 2 , 3 , 4 ). Some survivors exhibit persisting symptoms, partial recovery, and residual radiological abnormalities. Experiences with other coronaviruses, such as SARS and MERS studies, showed that fibrotic changes and opacities in the lung were detected in up to 36% of the individuals for months after infection ( 5 , 6 , 7 , 8 ). Some studies evaluating the third month results of patients who had COVID-19 showed that radiologic changes persisted in 25% to 63% ( 2 , 8 ).

This study aimed to evaluate the clinical, radiological, and pulmonary findings, as well as the quality of life in patients treated for COVID-19 three months after discharge. Both the functional status of the patients and the development of fibrotic changes in the lung were examined.

## MATERIALS and METHODS

At the beginning of the study, the minimum sample size was calculated as 54 with 95% confidence, effect size 0.5, and 95% power level through the G*power program.

**Figure 1:**
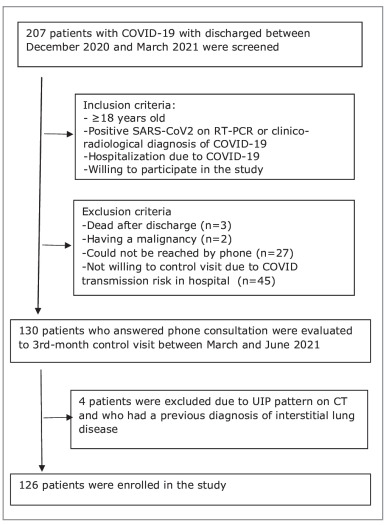
Flow chart of the study.

The study included patients who received inpatient treatment for COVID-19 and were discharged between December 2020 and March 2021. Admission as an acute inpatient with a positive SARS-CoV-2 nasopharyngeal swab on RT-PCR or a clinicoradiological diagnosis of COVID-19 was defined as COVID-19. The patients were offered a phone consultation with a respiratory physician three months after discharge. Patients who could not be reached by phone or who did not want to come for a follow-up visit (due to the risk of COVID transmission in the hospital) were excluded. The flow chart is given in Figure 1.

Inclusion criteria: -≥18 years of age -Positive SARS-CoV-2 on RT-PCR or clinico-radiological diagnosis of COVID-19 -Hospitalization due to COVID-19 -Willing to participate in the study Exclusion criteria: -Death after discharge -Having a malignancy -Could not be reached by phone -Not willing to come for a follow-up visit due to COVID transmission risk in the hospital -Previous diagnosis of interstitial lung disease

A total of 126 patients were enrolled in the study. Physical examination and clinical and radiological findings were evaluated at the follow-up visit. A pulmonary function test was performed on all patients using ZAN GPI 3.00 device (Nspire Health GmbH, Germany) at the third month follow-up visit. Symptoms during the illness and ongoing symptoms at the follow-up visit, comorbidities, and demographic information were recorded. Informed consent was obtained from all of the patients.

The Modified British Medical Research Council (mMRC) dyspnea scale was used for dyspnea assessment ( 9 ). A six-minute walking test was performed. The walking test could not be performed on two patients, one paraplegic and the other with walking difficulties.

The Turkish translation of St. George’s Respiratory Diseases Questionnaire (SGRQ), which has been validated in Turkish, was used for the quality-of-life assessment ( 10 ).

### Chest Computed Tomography (CT)

All CT images were independently reviewed by two chest radiologists with more than 10 years of experience, blinded to the clinical data and laboratory indicators, in a standard clinical image archiving and diagnostic system workstation. All thin-section CT images were reviewed at a window width and level of 1000 to 2000 HU and -700 to -500 HU, respectively, for lung parenchyma. After independent evaluation, the radiologists resolved any disagreement with discussion and consensus.

### Chest CT Procedure

Chest CT imaging was performed using a 64-detector CT scanner (Aquillon 64, Toshiba, Otawara, Japan). All patients were examined in the supine position. CT images were then acquired during a single inspiratory breath-hold. The scanning range was from the apex of the lung to the costophrenic angle. Scans were obtained in the craniocaudal direction without an iodine contrast agent. CT scan parameters were as follows: X-ray tube parameters 120 kVp; pitch 1.4; section thickness 1 mm; intersection space 0.8 mm. Images were sent to the workstation (Aquarius Intuition edition v4.4.6, TeraRecon, Foster City, CA, USA) for assessment. Reformatted images in sagittal and coronal planes with a slice thickness of 1 mm were constituted in addition to the axial plane with the same resolution characteristics. A low-dose computed tomography (CT) scan of the chest was used for the follow-up of patients.

### Chest Computed Tomography Severity Score

In this study, both lungs were divided into lobes and the lung opacities in all of the five lobes were evaluated on chest CT. Each lung lobe was assigned to one of the following categories based on the distribution of the affected lung parenchyma: 0, normal; 1, <25% abnormality; 2, 25-50%; 3, 50-75%abnormality; and 4, > 75% abnormality ( 5 , 11 ). The total severity score (TSS) was then reached by summing the points from each of the five lobes, which ranges from 0 to 20.

The radiologists assessed each of the five lobes of both lungs for the presence of inflammatory abnormalities, including the presence of groundglass opacities, mixed ground-glass opacities, consolidation predominant pattern, pleural or pericardial effusion, or fibrotic-like abnormalities (Linear opacities, traction bronchiectasis, honeycombing, reticulation) ( 12 ). When opacities are present, the distribution of the findings was graded according to their distribution (unilateral/ bilateral, involved lobes).

### Statistical Analysis

Kolmogorov-Smirnov, skewness, and kurtosis coefficients were used to test the normal distribution of the data. Normally distributed data were given as mean ± SD and non-normally distributed data as median (min-max). When comparing the first and third month data of the patients, paired t-test was used for normally distributed data, and the Wilcoxon test was used for those who did not show normal distribution. In independent two-group comparisons,the independent t-test was used if the data were normally distributed and the Mann-Whitney U test was used if the data were not normally distributed.Categorical data were expressed as numbers and percentages. Pearson Chi-square test was used for intergroup comparisons for categorical variables.Multivariable logistic regression analysis was performed to identify independent determinants that affect the fibrotic-like changes in the lung. A p value of <0.05 was considered significant in all analyses.SPSS 15.0 software (SPSS Inc., Chicago, IL) was used for statistical analysis.

### RESULTS

Demographic and clinical characteristics of the patients are given in Table 1. 60% of the patients were male and the mean age was 58.7 (± 12.9) years. The most common comorbidities were hypertension and diabetes mellitus (37.3% and 23.8%). The median duration of hospitalization was seven days [1-60 days (min-max)], 87% received supplemental oxygen and 15.0% received intensive care treatment. Patients with intensive care unit (ICU) hospitalization had a longer hospital stay [median 20 days (5-60 days, min-max)] (p< 0.001). There was no difference in age, sex, and comorbidities between those with and without ICU hospitalization.

**Figure 2:**
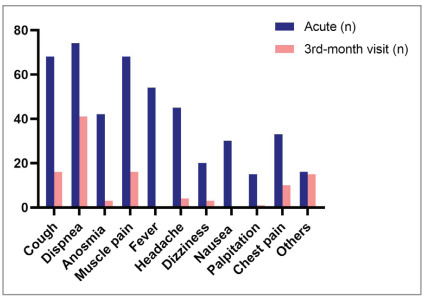
Symptoms of the patients during acute COVID-19 and follow-up visit.

The most common symptoms at initial presentation to the hospital were dyspnea (58.7%), cough (54%), muscle pain (54%), and fever (42.9%). At the third month follow-up visit, the most common symptoms were shortness of breath (32.5%), cough (12.7%), and muscle pain (12.7%) (Figure 2).

**Table 1:** Demographic features of the patients

Age	58.7 ± 12.9
Male sex, n (%)	76 (60.3)
Smoking history, n (%)	
Non-smoker	69 (54.8)
Smoker	57 (45.3)
Comorbidities, n (%)	
Any	42 (33.3)
Hypertension	47 (37.3)
Diabetes mellitus	30 (23.8)
Cardiovascular disease	26 (20.8)
COPD	9 (7.1)
Asthma	10 (7.9)
Chronic liver disease	2 (1.6)
Chronic kidney disease	1 (0.8)
Cerebrovascular disease	3 (2.4)
In-hospital treatment	
Length of stay (days)	7 (1-60)
Oxygen therapy, n (%)	110 (87.3)
ICU admission, n (%)	19 (15.0)
NIMV, n (%)	10 (7.9)
IMV, n (%)	5 (3.9)
HFO, n (%)	4 (3.1)

Table 2 shows the clinical evaluation outcomes from the third-month follow-up visit. In pulmonary function tests, the mean FVC and FEV_1_ values were 89% and 92%, respectively. Patients with ICU hospitalization had lower lung function capacities (FEV_1_%, FVC%, FEV_1_/FVC) than those without ICU hospitalization (p= 0.004, p= 0.027, p= 0.037, all antimicrobial categories.

**Table 2:** Lung function, exercise capacity and quality of life of the patients at third month follow-up visit

	ICU (n= 19)	No ICU (n=107)	p
FVC, L^a^	2.8 ± 1.1	3.1 ± 0.9	0.263
FVC, % of predicted ^a^	80 ± 19	91 ± 20	0.027
FEV1, L^a^	2.2 ± 1	2.6 ± 0.8	0.104
FEV_1_, % of predicted^a^	79 ± 22	93 ± 19	0.027
FEV_1_/FVC, %^a^	79 ± 14	83 ± 7	0.037
SpO_2_ at rest, %^b^	96 (67-98)	97 (84-99)	0.008
SpO_2_ after walking test, %^b^	94 (83-98)	97 (70-100)	0.002
Heart rate before walking test ^a^	88 ± 13	87 ± 17	0.816
Heart rate after walking test^a^	104 ± 15	100 ± 19	0.318
Six-minute walking distance, m^a^	334 ± 147	342 ± 136	0.429
SGRQ			
Symptom score^a^	26.3 ± 21	21.7 ±17.7	0.323
Symptom score^a^	26.3 ± 21	21.7 ±17.7	0.323
Impact score^a^	26.9 ± 29.6	20.3 ± 19.3	0.216
Activity score^a^	32.4 ± 31.8	26.3 ± 23.7	0.333
Total score^a^	28.3 ± 26.4	22.3 ± 18.5	0.227
mMRC≥ 1, n (%)	10 (52.6)	59 (55.1)	0.840

^a^: Normally distributed data presented as mean ± SD, independent-samples t-test. ^b^: Non-normally distributed data presented as median (min-max), Mann-Whitney U test

respectively). In 12 patients, the oxygen saturation level at rest was ≤93%. In 17 patients, oxygen saturations at rest were 94-95%. The remaining 97 patients had an oxygen saturation of 96% and above. At the third month follow-up visit, oxygen saturations at rest and after six-min walking were lower in patients with prior ICU admission compared to those without (p< 0.001, p= 0.004). Mean six-min walk distances were similar in the two groups (p= 0.429). Quality of life scores were lower in those who were hospitalized in the ICU than those who did not, yet the difference was not statistically significant.

The laboratory parameters of the patients at admission and the third month follow-up visit are given in Table 3. Neutrophil ratios were higher, and lymphocyte and eosinophil ratios were significantly lower during the acute disease period than at follow-up. When biochemical parameters were analyzed, urea, creatinine, liver enzymes, LDH, ferritin, troponin, and D-dimer were significantly higher in the acute disease period. The third month laboratory results were within the expected reference range.

**Figure 3:**
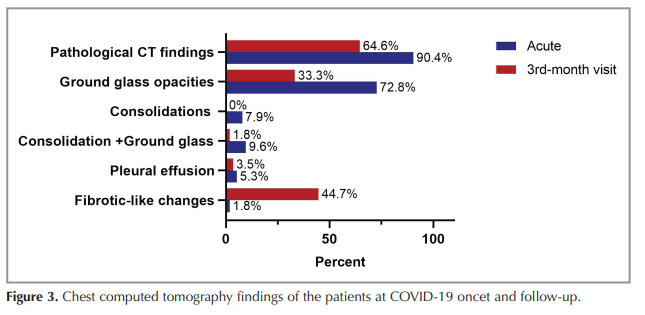
Chest computed tomography findings of the patients at COVID-19 oncet and follow-up.

**Figure 4:**
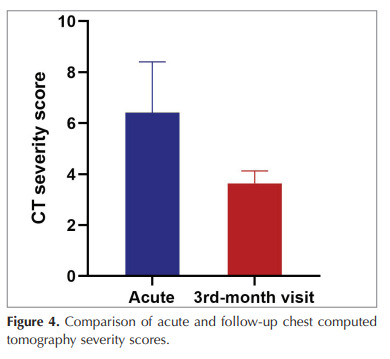
Comparison of acute and follow-up chest computed tomography severity scores.

Of the patients 90.4% had pathological radiological abnormalities during the acute disease period, while 64.6% had radiological findings at the three-month follow-up (Figure 3). Comparison of CT severity scores at admission and third month follow-up showed a significant improvement (7.8 ± 5.0 vs 3.3 ± 3.9, respectively, p< 0.001) (Figure 4). On the initial thorax CT, the rate of bilateral lung involvement was 86% and the rate of involvement of all lung lobes (five lobes) was 71.9%. The main thorax CT findings at admission were ground glass opacities (GGO) (72.8%), consolidation (7.9%), and consolidation + GGO (9.6%). At the third month follow-up, the most common findings on thorax CT were fibrotic-like changes in 44.2% and GGO in 33.3%.

**Figure 5:**
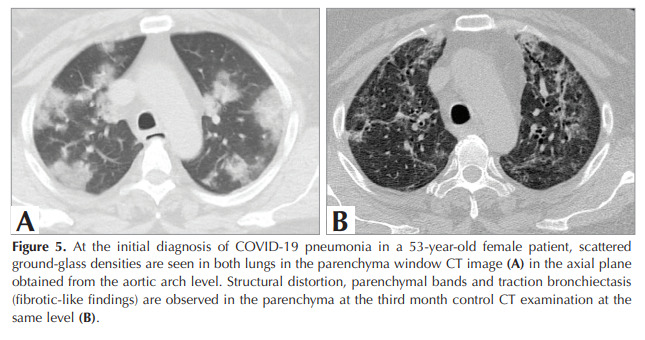
At the initial diagnosis of COVID-19 pneumonia in a 53-year-old female patient, scattered ground-glass densities are seen in both lungs in the parenchyma window CT image (A) in the axial plane obtained from the aortic arch level. Structural distortion, parenchymal bands and traction bronchiectasis (fibrotic-like findings) are observed in the parenchyma at the third month control CT examination at the same level (B).

**Figure 6:**
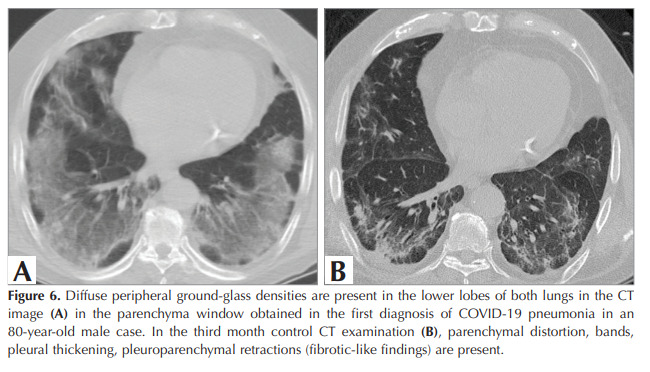
Diffuse peripheral ground-glass densities are present in the lower lobes of both lungs in the CT image (A) in the parenchyma window obtained in the first diagnosis of COVID-19 pneumonia in an 80-year-old male case. In the third month control CT examination (B), parenchymal distortion, bands, pleural thickening, pleuroparenchymal retractions (fibrotic-like findings) are present.

Patients with fibrotic-like changes (n= 50) were subdivided into bronchiectasis (n= 14), structural distortion (n= 23), reticulations (n= 27), and parenchymal bands (n= 33). As an example of fibrotic-like changes, CT images of two patients are presented in Figures 5A, 5B, and Figures 6A, 6B.

Fibrotic-like alterations were more common in men, those receiving intensive care, those with higher total CT severity, and those with more involved lobes (p< 0.05) (data not shown). There was no relationship between smoking status and fibrotic changes (p= 0.068).

Parameters that may be associated with fibrotic-like changes were evaluated by multivariable logistic

**Table 3:** Laboratory parameters of the patients during acute COVID-19 and follow-up

	Acute	Third month visit	p
White blood cell, (mm3) ^c^	7000 (1700-24700)	7600 (3400-15100)	0.634
Neutrophils, (mm3) ^c^	4680 (555-22200)	4400 (1600-12600)	0.034
Neutrophils, (%)^d^	71.2 ± 14.9	59.7 ± 10.9	<0.001
Lymphocytes, (mm3) ^c^	1200 (40-8760)	2100 (700-8800)	<0.001
Lymphocytes, (%)^d^	20.3 ± 14.8	29.7 ± 10.8	<0.001
Eosinophils, (mm3)^c^	5 (0-700)	100 (0-1200)	<0.001
Eosinophils, (%)^c^	0.4 (0.0-9.9)	1.8 (0.0-8.1)	<0.001
Hemoglobin, (g/dL)^d^	13.9 (± 7.2)	13.5 (± 1.7)	0.585
Hematocrit, (%)^d^	39.5 (± 5.2)	40.5 (± 4.6)	0.007
Platelet count, (mm3) ^d^	270155 (± 122052)	267909 (± 68955)	0.835
Urea, (mg/dL)^d^	38.8 ± 20.2	31.2 ± 9.5	<0.001
Creatinine, (mg/dL)^d^	0.97 ± 0.28	0.9 ± 0.14	0.001
Symptom scorea	26.3 ± 21	21.7 ±17.7	0.323
AST, (IU/L)^c^	28 (15-144)	18 (10-52)	0.001
ALT, (IU/L)^c^	26 (8-158)	18 (6-88)	<0.001
LDH, (U/L)^c^	278 (10-1591)	205.3 (± 66.5)	<0.001
C-reactive protein, (mg/L)^c^	54.9 (4-235)	3.0 (2.0-63.2)	<0.001
Ferritin, (µg/L)^c^	214 (5.5-2500)	28.7 (4-435)	<0.001
Troponin, (ng/L)^c^	5.0 (0.0-760)	2.8 (0.1-57.8)	0.001
D-dimer, (mg/L)^c^	391 (32-30553)	150 (85-1041)	<0.001

^c^ : Non-normally distributed data expressed as median (min-max), Wilcoxon test. ^d^: Normally distributed data expressed as mean ± SD, paired-samples t-test.

regression analysis. Parameters that were significant in the univariate analysis, such as age, sex, steroid use, intensive care unit stay, duration of hospitalization, admission CT severity score, consolidation, number of involved lobes, oxygen saturation, admission lymphocyte (%), neutrophils (%), ferritin, D-dimer, hemoglobin, hematocrit, LDH and CRP levels were included in the regression model.

Age [95% confidence interval (CI), 1.01 to 1.15;p= 0.020], male sex (95% CI, 4.06 to 95.3, p< 0.001), first CT severity score (95% CI, 1.02 to 1.41,p= 0.028), duration of hospitalization (95% CI, 1.02 to 1.18, p= 0.012), oxygen saturation (95% CI, 0.86 to 0.96, p< 0.001) were independent predictors of fibrotic-like changes (Table 4).

### DISCUSSION

While COVID-19 continues to cause acute disease due to variants, the persistence of disease symptoms for an extended period of time (>3 months), referred to as post-COVID or long COVID, is still being studied. A study conducted in the USA reported that 24% of those with mild disease severity still had at least one symptom after 90 days, while this rate reached 40.6% in severe COVID-19 cases ( 13 , 14 ). A prospective cohort study conducted in Europe showed that dyspnea persisted in approximately half of the patients at the third month ( 2 ). In our study, 32.5% of the patients still had dyspnea. The second most common symptom was cough and muscle pain. Many studies describe the most common post-COVID symptoms as shortness of breath and cough ( 15 , 16 , 17 ).

**Table 4:** Independent predictors for the fibrotic-like changes according to regression analysis

	β	p	OR (95% CI)
Age	0.076	0.020	1.08 (1.01-1.15)
Male sex	2.979	<0.001	0.034
First CT severity score	0.184	0.028	1.20 (1.02-1.41)
SpO2 at rest	-0.093	<0.001	0.91 (0.86-0.96)
Duration of hospitalization	0.091	0.012	1.09 (1.02-1.18)

R2= 0.611, SpO2: Oxygen saturation. d: Normally distributed data expressed as mean ± SD, paired-samples t-test.

As in previous coronavirus pneumonias, decreased lung function and exercise capacity have been reported after COVID-19 ( 2 , 17 , 18 , 19 ).

In our study, exercise capacity and mMRC dyspnea scores were similar between ICU and non-ICU participants. However, patients with prior ICU admission have reduced lung capacity and they have more desaturation at rest and after six-min walking tests, compared to those without. Furthermore, SGRQ scores for quality of life assessment suggested that quality of life declined in those admitted to ICU, i.e. those with severe disease.

The main thorax CT findings in acute COVID-19 have been reported as bilateral, peripheral, GGO, and consolidation ( 5 , 20 ). In a multicenter study conducted in Europe, it was shown that residual radiologic changes, especially GGO, and reticulation, were present in 2/3 of the patients more than 100 days after the onset of the disease ( 5 ). In our study, GGO was observed in 72.6% of patients at the first hospital admission and 86% were bilateral.

It is predicted that GGO reflects acute inflammation ( 12 , 21 ). While GGO and consolidation heal slowly, fibrosis may develop in 50-60% of cases ( 12 , 22 , 23 ). A six-month follow-up study of 114 COVID-19 survivors revealed that 35% exhibited fibrotic-like changes ( 24 ). Previous studies have shown that some ARDS survivors develop fibrotic-like changes. It has been suggested that fibrotic-like changes in ARDS patients may be due to ventilator-induced lung injury. Because the number of survivors who received intensive care and were intubated in our study was limited, we believe that many other mechanisms other than ventilator injury may have contributed to this process.

In a three-month follow-up study of 52 COVID-19 survivors, 25% of whom had abnormal CT findings and 25% had never been hospitalized, 42% of patients were shown to have residual abnormalities ( 25 ). Although some of our cases were short-term, all of them were hospitalized and at the third month follow-up, 44.2% had fibrotic-like changes and 1/3 had GGO.

The term fibrosis, which is frequently used after COVID-19, is used for findings including parenchymal bands, subpleural bands, focal atelectasis, and reticular abnormalities ( 12 ). However, these changes may resolve completely in the long term.

The most commonly reported findings are parenchymal bands, regular interfaces, reticular patterns, and bronchial dilatation ( 20 , 21 , 26 , 27 ). Honeycomb development is very rare and has been described only in a few cases ( 28 ). In fact, honeycomb, traction bronchiectasis or bronchiectasis, and architectonic distortion are more specific findings of fibrosis ( 29 ). In our study, parenchymal bands were the most common finding in fibrotic-like changes followed by structural distortion and reticulations.

Certain factors have been identified as predictors in the development of fibrosis such as advanced age, high CRP and IL-6, prolonged hospitalization, and more involvement on thorax CT ( 26 , 30 ). In our series, in multivariable regression analysis, male sex was the most predictive factor for fibrosis, followed by age, baseline CT severity score, oxygen saturation, and duration of hospitalization.

There have been very few long-term (one year or more) follow-up studies on COVID-19. In this study, we obtained data for the third month therefore, we do not know the long-term effects. In a study of 71 SARS cases that were followed up for 15 years, abnormal CT findings (GGO and band-like consolidation) were found in 38% ( 31 ). The researchers showed that the rate of lung involvement decreased significantly in the first year and remained stable for the next 14 years.

The majority of patients did not have baseline thorax CT examinations before COVID-19, which is one of our study’s limitations. Furthermore, there is a lack of prolonged follow-up, such as six months or a year.

### CONCLUSION

Our data show that dyspnea is the most prevalent symptom three months after COVID-19, and the most common radiological findings are fibrotic-like changes and GGO, with the male sex being the greatest predictor factor for fibrosis. Longer follow-up studies are required to identify the long-term effects of COVID-19.

#### Acknowledgements

The authors thank Dr. Ezgi Türk from Balıkesir University Faculty of Medicine, Department of Public Health for her assistance in the statistical analysis.

##### Ethical Committee Approval

This study was approved by Balıkesir University Faculty of Medicine Clinical Research Ethics Committee (Decision no: 2021/63, Date: 10.03.2021)

#### CONFLICT of INTEREST

The authors declare that they have no conflict of interest.

#### AUTHORSHIP CONTRIBUTIONS

Concept/Design: NS, GD, HÇ, FE, GDA

Analysis/Interpretation: NS, GD, EB, ATA

Data acqusition: : NS, GDA, ATA, HÇ

Writing: NS, GD, GDA, EB, ATA

Clinical Revision: HÇ, FE, EB

Final Approval: NS, FE
